# Workload analysis of pilot steep turn maneuvers using SR20 aircraft and EEG data

**DOI:** 10.3389/fnins.2026.1721962

**Published:** 2026-05-25

**Authors:** Jiajun Yuan, Shihan Luo, Chenyang Zhang, Haotian Qiao, Hua Chen, Chaozhe Jiang

**Affiliations:** 1 School of Transportation and Logistics, Southwest Jiaotong University, Chengdu, China; 2 Department of Clinical Neurosciences, University of Calgary, Calgary, AB, Canada; 3 Key Laboratory of Transport Industry of Management, Control and Cycle Repair Technology for Traffic Network Facilities in Ecological Security Barrier Area, Chang'an University, Xi'an, China; 4 Tangshan Institute, Southwest Jiaotong University, Tangshan, China

**Keywords:** EEG, machine learning, pilot, steep turn, workload

## Abstract

**Objective:**

To compare left vs. right steep turns in terms of workload-related neurophysiological signatures using electroencephalogram (EEG) and machine learning.

**Methods:**

Thirty-seven flight cadets performed one left and one right steep turn in an SR20 desktop flight simulator while a 32-channel EEG (Emotiv EPOC Flex 32) was recorded. From 2-s sliding windows (50% overlap), 800 features per window were extracted (time-, frequency-, and non-linear domains). Six classifiers (XGBoost, LightGBM, GB, SVM, LR, and Linear SVC) were evaluated using cross-subject nested cross-validation with variance-ranked feature subsets (20%, 40%, 60%, 80%, and 100%), and an additional 10% subset was assessed to identify a more parsimonious feature set.

**Results: Objective EEG/ML:**

LightGBM demonstrated superior performance across all feature proportions.

**Subjective:**

NASA-TLX was significantly higher in right turns than in left turns (5.55 ± 1.13 vs. 4.98 ± 1.06, *p* < 0.001, and Cohen's *d* = 0.52). *Post-hoc* interpretation combined RF-based importance and variance-ranked top-feature analysis, showing convergent frontal/frontocentral dominance with complementary utility definitions (predictive contribution vs. signal dispersion). Physiologically, left turns were associated with relatively higher high-frequency activity/complexity, whereas right turns showed relatively stronger theta/alpha-related patterns.

**Interpretation:**

These findings support MWL-associated directional neurophysiological differences in steep turns and identify candidate EEG markers for lightweight real-time workload monitoring, facilitating optimized flight training and enhanced aviation safety.

## 1 Introduction

Turn maneuvers include steep turns, ground reference maneuvers, turns during slow flight, and instrument flight turns (Federal Aviation Administration, 2016), constituting a core component of private pilot license (PPL) training and representing a comprehensive demonstration of pilots' safety control, navigation capabilities, and emergency response skills. Turns are classified by bank angle as shallow, medium, or steep, with steep turns defined as those with bank angles of 45° or greater. In such situations, pilots require more precise and coordinated control inputs, including rudder to maintain coordination, elevator to maintain altitude, and ailerons to counteract overbanking, while simultaneously monitoring aircraft instruments and the external environment, resulting in a significantly increased workload. Research indicates that pilot error accounts for 58% of fatal aviation accidents (Yang et al., 2021), with excessive workload being a primary contributing factor. High workload may lead to pilot distraction, loss of situational awareness, and even operational errors (Idowu and Shogbonyo, 2022), resulting in serious aviation accidents. Therefore, reasonable management and control of pilot workload during steep-turn maneuvers are key measures to ensure flight safety.

Mental workload (MWL) refers to the cognitive resources consumed by information processing, decision-making, and situational awareness during flight, serving as the cognitively dominant core of the integrated task demand (which also includes motor execution and perceptual processing). Pilot workload assessment uses multiple methodological approaches (Wang et al., 2025), including subjective assessment, behavioral performance evaluation, and physiological signal monitoring. Physiological signal monitoring represents a more sophisticated approach that uses biomarkers such as heart rate variability, electrodermal activity, oculomotor patterns, and electroencephalography (EEG) to evaluate workload. This methodology offers the distinct advantage of real-time, objective assessment of pilots' physiological and cognitive states, though individual physiological parameters vary in sensitivity and specificity in workload detection. Among these approaches, EEG emerges as a particularly promising non-invasive neurophysiological technique for workload assessment. EEG provides direct measurement of cortical electrical activity with exceptional temporal resolution, enabling real-time quantification of fluctuations in cognitive load (van Weelden et al., 2022). Unlike peripheral physiological measures, EEG signals represent direct neural correlates of cognitive processing, facilitating precise detection of workload variations during complex flight operations. Specifically, power spectral changes in the theta and alpha frequency bands show robust correlations with MWL, whereas higher-frequency components, including beta and gamma oscillations, reflect attentional engagement and sensorimotor integration processes. Consequently, EEG-based assessment offers substantial potential for advancing pilot workload evaluation methodologies.

EEG-based research on pilot workload has predominantly focused on simulated flight environments. Liu et al. (2024) employed low-intrusion EEG devices to collect physiological data from pilots during simulated flight tasks, proposing a real-time pilot workload detection model based on EEG signals by combining k-nearest neighbor (KNN) algorithms and ensemble learning methods, achieving a detection accuracy of 82.6% on cross-pilot test sets. van Weelden et al. (2024) collected EEG and behavioral data from six military pilots (men) performing high and low workload tasks in a virtual reality flight simulator, finding that theta-band EEG power was a key feature, with baseline-corrected EEG feature-based classifiers performing optimally. Lee et al. (2020) induced fatigue, workload, distraction, and normal states in seven pilots through simulated flight experiments. While collecting EEG data, they proposed a multi-feature block convolutional neural network (MFB-CNN) model for real-time decoding of pilots' current mental states, achieving 75% classification accuracy for four mental states. Moreover, Lee et al. (2024) induced normal, low workload, and high workload states through simulated flight experiments, collecting EEG data from 10 pilots, and proposed a hybrid deep neural network model containing five convolutional blocks and one long short-term memory unit, achieving an average accuracy of 86.13%. Furthermore, Liu et al. (2023) obtained EEG data from 21 pilots completing traffic pattern flights, using various machine learning algorithms and Bayesian neural networks for classification. They found that KNN models combined with all power spectral density (PSD) features achieved the highest accuracy. Hernández-Sabaté et al. (2024) collected pilot EEG data in flight simulators using Emotiv Epoc X EEG devices, which analyzed EEG features through deep learning models to predict pilot workload, achieving 76.25% accuracy after training on N-back test data. Causse et al. (2015) collected pilot EEG data and flight performance during simulated flight tasks, and found that high workload led to decreased pilot task performance and reduced P3b wave amplitude in EEG signals. Seedhouse (2024) studied the cognitive performance of four pilots during standard and emergency simulated flights in day and night conditions; they found significantly increased theta band power in high-load tasks. Feltman et al. (2024) collected EEG data from eight Army pilots during simulated flight in UH60 Black Hawk helicopter full-motion simulators, which significantly decreased frontal alpha and theta bands in EEG data under high load conditions. Singh et al. (2021) used EEG data from 14 pilots during human-machine collaborative tasks, which showed significantly increased EEG beta and gamma band power under high workload conditions. Hernández-Sabaté et al. (2022) collected pilot EEG data during low, medium, and high workload tasks in flight training devices, which successfully distinguished different workload levels using convolutional neural network (CNN) models. A limited number of existing studies have been conducted based on actual flight operations. Wilson et al. (2021) analyzed EEG data from 10 FAA commercial pilot license holders performing high and low workload simulated flight tasks, which distinguished between high and low workload using support vector machine (SVM) models with 95% accuracy. Dehais et al. (2019) collected pilot EEG data under real flight conditions, finding higher P300 amplitudes and stronger alpha and theta band power under low workload conditions, with single-trial classification based on frequency features achieving 70.8% accuracy. Taheri Gorji et al. (2023) collected EEG data from 10 pilots during flight, which extracted features through PSD and logarithmic energy entropy analysis. They used predictive models based on recursive feature elimination and stacked ensemble machine learning algorithms to distinguish among low, medium, and high workloads across different flight phases, such as turns, achieving a model accuracy of 91.67%.

Although existing studies have analyzed pilot workload using EEG signals, most research has focused on specific flight phases or task types, such as takeoff, cruise, and landing phases. There is relatively little research on left and right turns during steep banking maneuvers. Heath (2003) compared different scoring methods for level-turn tasks in flight simulators, finding that automated scoring correlated with instructor scoring but showed notable differences. Ji et al. (2024) analyzed pilot brain activity during left and right turns using EEG feature analysis, finding that β-wave energy and Shannon entropy during turning phases were significantly higher than during cruise phases, achieving a test-set accuracy of 93.67% using SVM. Zhang et al. (2022) measured changes in brain activity of 25 pilots during left and right turns using flight simulators and fNIRS, finding that different turning behaviors were associated with the frontopolar cortex (BA10). Yuan et al. (2024) measured physiological signal changes in pilots during left and right turn tasks and analyzed oxyhemoglobin concentration changes through machine learning models, discovering that different turning behaviors were closely related to brain cortical activities in BA17, BA18, and BA46 regions, with the SVM-RBF classification model achieving 92.6% accuracy in identifying pilot turning behaviors. Zhou et al. (2024) collected heart rate variability data from 28 pilots during climbing, level, and descending turns using flight simulators and heart rate sensors, finding that the LSTM-Attention model achieved 94.91% accuracy in recognizing cognitive load across different turning tasks. However, existing turning-related studies primarily focus on discriminating physiological signals across maneuver types, rather than explicitly quantifying differences in MWL magnitude between left and right steep turns. Moreover, these studies rarely disentangle directional MWL effects from co-varying task factors such as control-input asymmetry, procedural familiarity, and visual-reference constraints. Therefore, whether directional maneuver asymmetry induces distinct MWL profiles and whether such profiles can be robustly mapped to physiological features remain open questions. Beyond aviation, directional asymmetry has been reported in other human–machine control contexts (e.g., road-traffic turning maneuvers; Hancock et al., 1990), where left–right tasks can impose unequal workload (left-turn = 27.58, right-turn = 34.29). While such evidence is not directly transferable to flight operations, it supports the broader plausibility that directional maneuvering may induce asymmetric cognitive workload. In aviation, this plausibility is further strengthened by flight operations doctrine and handling characteristics: left and right turns are operationally comparable but not fully symmetric in control requirements, due to differences in propulsion-related effects, coordination demands, and visual-reference usage (Federal Aviation Administration, 2022). Therefore, left–right steep turns provide a theoretically grounded and operationally relevant paradigm for testing whether directional maneuver asymmetry is associated with measurable differences in MWL.

Although prior studies have explored physiological differences across turning behaviors, three gaps remain. First, limited direct quantification of MWL magnitude differences specifically between left and right steep turns. Second, insufficient discussion of how directional asymmetry may interact with potential confounding factors (e.g., control effort, familiarity, and visual-reference constraints) to shape MWL. Third, limited evidence on whether MWL-related neural patterns under directional maneuvers can be robustly mapped by machine-learning models. To address these gaps, the present study uses a within-subject left–right steep-turn paradigm, combines subjective workload assessment with EEG-based analysis, and evaluates whether discriminative physiological features can reliably classify MWL states under directional maneuvering conditions.

## 2 Materials and methods

### 2.1 Participants

A priori power analysis was conducted using G^*^Power 3.1.9.7 before data collection. The test was specified as a *t*-test → Means: Difference between two dependent means (matched pairs), with a two-tailed α = 0.05, desired power (1–β) = 0.80, and a medium expected effect size (Cohen's dz = 0.50). The analysis indicated that a minimum of 34 paired observations would be required to achieve 80% power. The final sample included 37 participants, exceeding the required minimum. Under the same assumptions, the achieved *post-hoc* power was approximately 1–β ≈ 0.84, demonstrating adequate statistical power to detect effects of medium magnitude or larger in within-subject comparisons.

Thirty-seven healthy adult men right-handed flight cadets from the Civil Aviation Flight University of China (CAFUC) were recruited as participants (age 23.65 ± 0.79 years, height 179.86 ± 5.13 cm, weight 71.19 ± 10.39 kg, 245 ± 10 flight h). All participants were men and right-handed, reflecting the composition of the eligible training cohort at CAFUC during the study period, as the flight training program enrolled only men cadets. All participants had completed flight training on SR20 aircraft and obtained PPL licenses. All participants had no color blindness or color weakness, possessed normal vision and hearing, had no history of heart disease, and held Class I medical certificates required by the Civil Aviation Administration of China. No participants consumed alcohol, coffee, or medications within 24 h before the experiment. The experiment complied with the Declaration of Helsinki, established by the World Medical Association, and was approved by the Ethics Committee of the CAFUC (No. 2024-7). All participants read and signed informed consent forms before the experiment and received appropriate compensation.

### 2.2 Flight simulator

**Figure 1:**
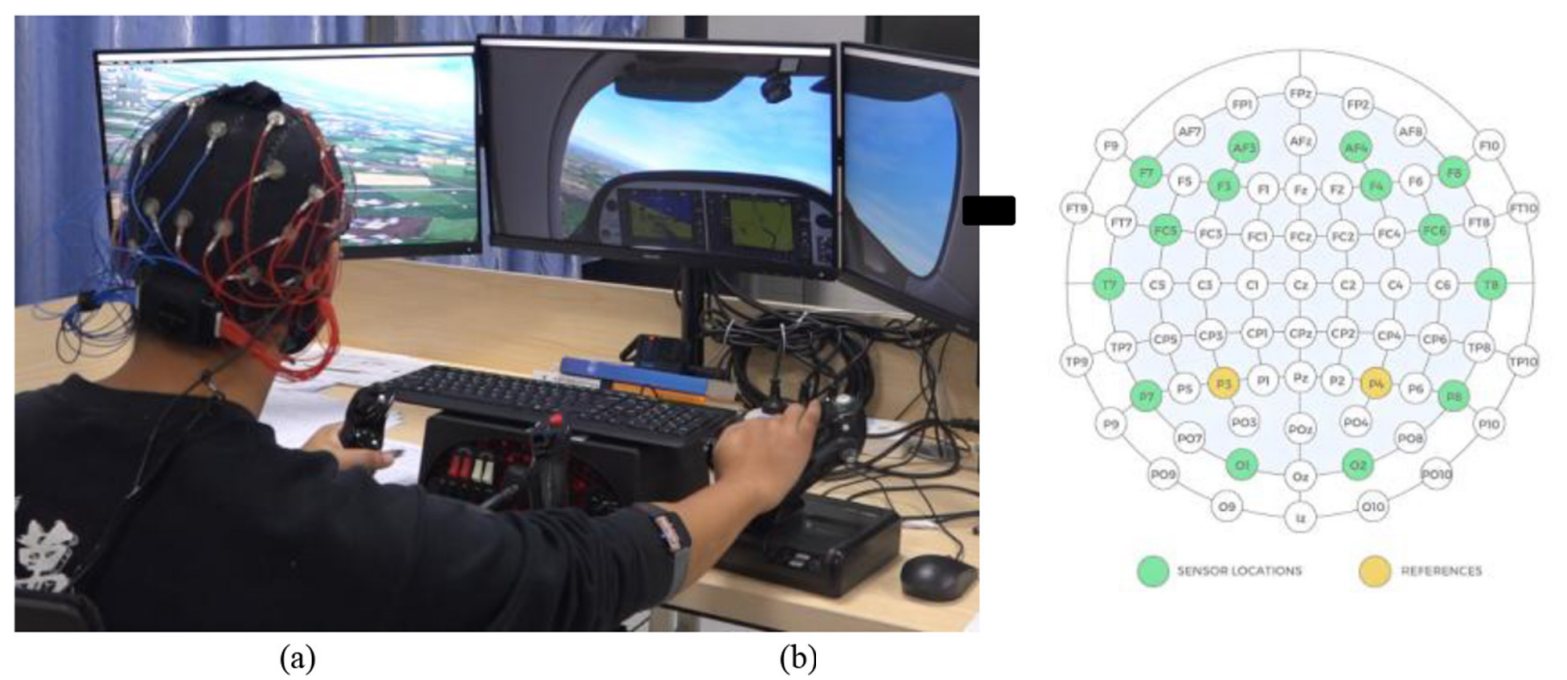
SR20 aircraft and desktop flight simulation equipment. **(a)** Flight simulator and **(b)** EEG channel location of the 10**–**20 system.

The aircraft model used in the experiment was the Cirrus SR20 single-engine propeller aircraft. This aircraft, with its safety, reliability, economy, and ease of operation (Dahlström et al., 2006) has become one of the most-produced and widely used civilian aircraft globally, serving as the primary trainer of choice for flight schools worldwide and extensively used for PPL training. The experiment was conducted on a desktop flight training device at the CAFUC, as shown in Figure 1a. This device was equipped with Microsoft Flight Simulator 2020 software, which integrates Bing Maps satellite data with real-time weather systems to provide an immersive cockpit perspective and simulated flight experience. The control equipment consisted of a Honeycomb YOKE multi-function control yoke, Logitech Flight Rudder Pedals, and Logitech X52 Pro throttle quadrant, all of which functioned normally and were compatible with the flight simulation software. The desktop trainer was capable of simulating the flight characteristics of the SR20 aircraft, providing participants with a virtual flight training environment that offered realistic flight conditions and operational experiences, and presenting operational skills and the ability to respond to complex weather conditions and emergencies encountered in actual flight.

The Emotiv EPOC Flex 32 (gel electrodes, 10**–**20 electrode placement standard) was used for EEG data acquisition device, as shown in Figure 1b. This is a high-density, wireless, and portable EEG system with a 32-channel layout that covers key brain regions. The device incorporates a built-in nine-axis inertial measurement unit sensor for real-time head movement tracking, with a sampling rate of 256 Hz. As a high-end device designed for neuroscience and brain-computer interface (BCI) applications (Williams et al., 2020), it can capture rich information about brain activity, providing precise and efficient data support for complex research tasks and various application scenarios.

### 2.3 Experimental tasks

**Figure 2:**
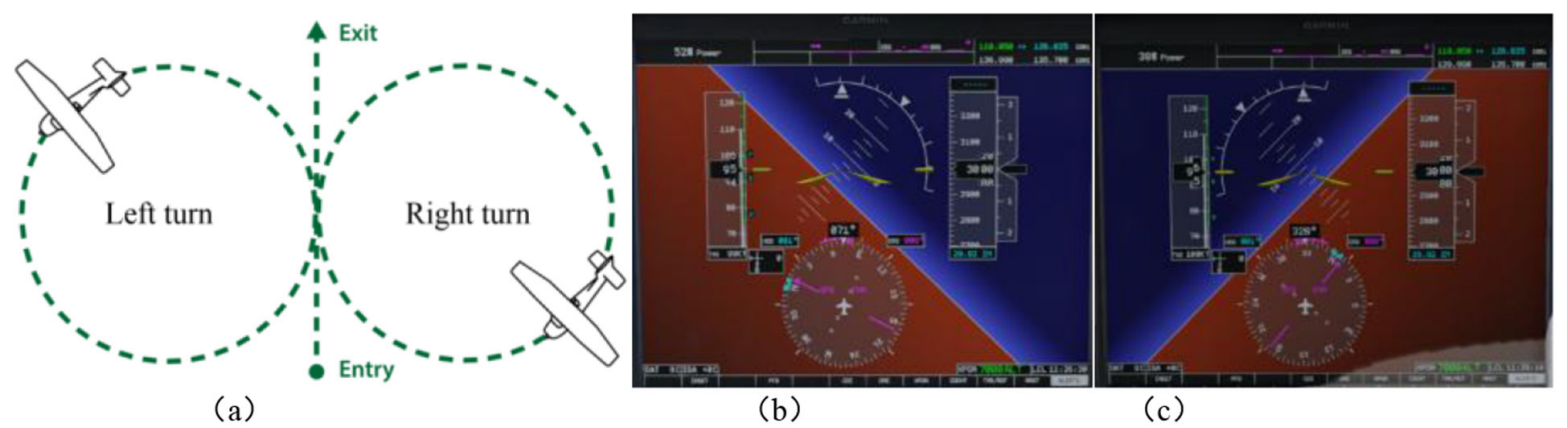
Steep turns of the SR20 aircraft. **(a)** Steep turns, **(b)** 45° left-turn, and **(c)** 45° right-turn.

Steep turns refer to turning maneuvers in which the aircraft achieves a bank angle of 45° or greater. The process requires pilots to precisely coordinate aileron, rudder, and elevator operations^1^ while maintaining relatively stable altitude and airspeed to complete large-angle banked turns. This is a key subject for assessing pilots' flight-control precision, situational awareness, and multitasking coordination. The experiment selected Runway 13 at Guanghan airport as the departure location, with weather conditions set to clear skies with no wind or light wind. Due to constraints of the institutional training protocol, the experimental task followed a fixed sequence: first, performing one complete steep turn to the left, followed by one complete turn to the right. This sequence aligns with CAFUC's standard flight training procedure for steep turns, which prioritizes left turns to leverage the natural left-roll torque tendency of single-engine propeller aircraft and reduce initial training difficulty. While we acknowledge the potential for order effects and fatigue, this fixed sequence was unavoidable given the need to adhere to official training guidelines to ensure ecological validity of the task. The standard experimental task consisted of performing one complete steep turn to the left first, followed by one complete turn to the right, as shown in Figure 2a. The specific implementation procedure was as follows:

1. Take off from Guanghan Runway 13 on downwind leg heading 127°, target altitude 3,000 ft.
2. After climbing to 1,800 ft altitude, maintain leg downwind heading 127° and continue climbing to 3,000 ft.
3. Upon reaching the target altitude of 3,000 ft, level off and maintain level flight heading and altitude, stabilizing the aircraft state. Confirm entry into heading 127° and altitude 3,000 ft.
4. Maintain 95 kt straight and level flight at approximately 2,300 RPM cruise power. Select an appropriate heading and smoothly increase the bank angle to enter a left-turn, targeting a 45° bank angle.
5. As bank angle increases, apply back pressure on the controls to maintain altitude and appropriately increase power to maintain airspeed. During the turn, maintain consistent bank angle, altitude, and speed, as shown in Figure 2b.
6. Recovery: (a) Before reaching the desired heading/reference point, smoothly level the bank angle and maintain straight and level flight. (b) Release the back pressure applied during the turn to maintain altitude. (c) Reduce power to pre-entry settings.
7. After confirming clear airspace, immediately perform a 360° turn in the opposite direction to the right, as shown in Figure 2c. After completing the second turn, return to straight and level cruise flight, ending the experiment.

The operational sequence of performing steep turns “left first, then right” represents a scientifically grounded choice established within the global civil aviation training system, based on aircraft characteristics, human factors engineering, and training safety considerations. This sequence leverages the natural left-roll torque tendency of single-engine propeller aircraft to reduce the difficulty of left turns, thereby providing a foundation for torque compensation during subsequent right turns. It also aligns with the left-seat cockpit layout, which offered superior visibility and ergonomic advantages that support a “simple-to-complex” training logic and mitigate the risk of situational awareness loss. Furthermore, prioritizing the left-turn helps prevent right-turn-related issues, such as excessive bank angles or sideslip, while maintaining procedural consistency, thereby enhancing skill transferability and ensuring fair evaluation during training and assessment.

### 2.4 Experimental procedure

Thirty-seven flight students who had completed SR20 flight training were invited to participate. Before the experiment began, participants were given 15 min to familiarize themselves with the flight simulator and review the experimental tasks by studying the SR20 pilot training manual. Then, participants completed a “Participant Experimental Information Form” to collect basic information and flight history, including prior flight-hour breakdown by maneuver type (left vs. right steep turns) to quantify procedural familiarity. During this period, participants wore the EEG acquisition equipment, and data transmission was verified to be functioning normally. Subsequently, experimental personnel confirmed that all preparatory work had been completed. After a 5-min rest period, participants took off from Guanghan Runway 13, climbed to the designated altitude, and completed the steep turn maneuvers. The specific timing of each participant's task performance was recorded to facilitate subsequent data analysis. After each experiment, experimental personnel extracted and labeled the EEG data, ensuring that each participant's data was properly matched and valid.

## 3 Data processing

### 3.1 Data preprocessing

A systematic EEG signal preprocessing pipeline was implemented using MNE-Python and additional libraries, including autoreject and scipy. The preprocessing steps included automatic detection and interpolation of bad channels using z-score thresholding (threshold = 3.5), band-pass filtering (1–45 Hz, implemented with a FIR filter of 128 taps, forward–backward zero-phase filtering, corresponding to ~0.25 s group delay), and notch filtering (50 Hz notch, FIR design consistent with 256 Hz sampling rate) to eliminate baseline drift and power line noise. Independent component analysis (ICA) with 95% variance retention to identify and remove electrooculography (Pérez et al., 2024) artifacts, using Fp1 and Fp2 as reference channels and re-referencing to the average brain potential. Additionally, an autoreject algorithm was implemented with individualized thresholds to exclude epochs containing significant artifacts. For validation, we compared the PSD of representative channels before and after filtering, confirming the effectiveness of drift removal and suppression of power-line interference. The implementation of these methods ensured the accuracy and reliability of EEG data, providing a high-quality dataset for further analysis.

### 3.2 Feature extraction

Feature extraction and machine-learning modeling were performed at the window level. Specifically, each participant completed one left-turn and one right-turn maneuver (each considered a trial). Within each trial, EEG was segmented using 2-s sliding windows (Xiao and Bo, 2024) with 50% overlap (Salimpour et al., 2022), and each window constituted a sample for subsequent classification. For every window, 800 features were extracted from 32 channels (9 time-domain, 10 frequency-domain, and 6 non-linear features; Table 1). After window-level feature extraction, all windows from all subjects and all trials were pooled to form the final dataset used for model training and evaluation. To prevent information leakage due to overlapping windows or subject-specific EEG characteristics, cross-validation was performed in a subject-independent grouped manner (StratifiedGroupKFold), ensuring that all windows from the same subject were assigned exclusively to either the training or test set in each fold. Both feature extraction and classification were performed at the trial level, with each trial representing a complete EEG segment from a left or right steep-turn task. All trial data were loaded from two independent files, labeled, merged, column-aligned, and numerically processed before feature extraction. Time-domain feature extraction was based on the amplitude statistical characteristics and dynamic variation patterns of signals, implemented through the extract_time_domain_features function. Frequency-domain feature extraction was based on the frequency-specific patterns of EEG signals and was implemented through the extract_frequency_domain_features function. Non-linear feature extraction was based on the non-linear dynamics of EEG signals and implemented through the extract_non-linear_features function. Furthermore, for frequency-domain feature extraction, the PSD was computed using Welch's method (Angkan et al., 2024) to estimate the signal's power spectral density, which converted time-domain signals into a frequency-domain energy distribution and reduced the variance of spectral estimation compared to a direct FFT. Based on the widely accepted EEG frequency band divisions in neuroscience research, the absolute power of each frequency band (total energy within the frequency band) was calculated by integration (Simpson's method), reflecting the activity intensity of that frequency band. Relative power (the ratio of absolute power in a specific frequency band to total power) was calculated to eliminate interference from individual amplitude differences and enhance feature comparability.

**Table 1:** EEG features and their descriptions.

Domain	Feature	Description
Time	Mean	The mean of the signal is a statistic that describes the central tendency of the signal data.
	Var	The variance of the signal is a statistic describing the degree of deviation of the signal data from the mean (the square of the standard deviation).
	Std	The standard deviation of the signal is a statistic describing the degree of dispersion of the signal data (the square root of the variance).
	Ptp	The peak-to-peak value of the signal, the difference between its maximum and minimum values, reflects its amplitude range.
	Rms	The root mean square (RMS) of the signal, calculated as the square root of the mean of the squared signal, is used to measure the effective amplitude of the signal.
	Zcr	The zero-crossing rate (ZCR) of a signal, the number of times the signal crosses the zero value per unit time, is used to describe the frequency characteristics of the signal.
	Skew	Skewness of a signal is a statistic that describes the degree of asymmetry of the signal data distribution.
	Kurt	The kurtosis of the signal, a statistic describing the peakedness of the signal data distribution, measures the tail thickness and peak height of the distribution.
	Hjorth_activity	Hjorth activity of the signal, reflecting the energy or amplitude changes of the signal.
Frequency	Delta_abs_power	Absolute power of the Delta band (0.5–4 Hz), i.e., the actual power value of the Delta band.
	Delta_rel_power	Relative power of the Delta band, i.e., the percentage of the Delta band power relative to the total power.
	Theta_abs_power	Absolute power of the Theta band (4–8 Hz), i.e., the actual power value of the Theta band.
	Theta_rel_power	Relative power of the Theta band, i.e., the percentage of the Theta band power relative to the total power.
	Alpha_abs_power	Absolute power of the Alpha band (8–13 Hz), i.e., the actual power value of the Alpha band.
	Alpha_rel_power	Relative power of the Alpha band, i.e., the percentage of the Alpha band power relative to the total power.
	Beta_abs_power	Absolute power of the Beta band (13–30 Hz), i.e., the actual power value of the Beta band.
	Beta_rel_power	Relative power of the Beta band, i.e., the percentage of the Beta band power relative to the total power.
	Gamma_abs_power	Absolute power of the Gamma band (30–100 Hz), i.e., the actual power value of the Gamma band.
	Gamma_rel_power	Relative power of the Gamma band, i.e., the percentage of the Gamma band power relative to the total power.
Nonlinear	Samp_entropy	Sample entropy of the signal, an indicator measuring the complexity of the signal time series; a larger value indicates lower regularity and higher complexity of the signal.
	Perm_entropy	Permutation entropy of the signal, a complexity metric based on element arrangement patterns in the signal sequence, is used to evaluate the randomness or regularity of the signal.
	Svd_entropy	SVD entropy (Singular Value Decomposition entropy) of the signal, an entropy metric based on singular value decomposition, which quantifies complexity through the distribution of singular values of the signal matrix.
	Katz_fd	Katz's fractal dimension of the signal describes the fractal characteristics of the signal time series and reflects the complexity and degree of tortuosity of the signal.
	Hjorth_mobility	Hjorth mobility of the signal, reflecting the speed of signal changes (the ratio of the standard deviation of the first derivative to the standard deviation of the signal itself).
	Hjorth_complexity	Hjorth complexity of the signal, reflecting the complexity of the signal waveform (the ratio of the standard deviation of the second derivative to the standard deviation of the first derivative).

### 3.3 Machine learning algorithms

XGBoost (extreme gradient boosting)

XGBoost is an efficient gradient boosting framework designed for classification and regression tasks. It optimizes the gradient boosting algorithm with parallel processing capabilities, automatic handling of missing values, and regularization terms to prevent overfitting (Khan et al., 2024). The model objective function is shown in Equation 1.


$$\begin{array}{l} L\;=\sum_{i\;=\;1}^{n}l\left(y_{i},\;{\overset{ˆ}{y}}_{i}\right)+\sum_{k\;=\;1}^{K}\Omega \left(f_{k}\right) \end{array}$$ (1)

Where $l\left(y_{i},{\overset{ˆ}{y}}_{i}\right)$ is the loss function and Ω(*f*_*k*_) is the regularization term used to control model complexity, as shown in Equation 2.


$$\begin{array}{l} \Omega \left(f_{k}\right)\;=\;\gamma T\;+\;\frac{1}{2}\lambda \sum_{j\;=\;1}^{T}w_{j}^{2} \end{array}$$ (2)

Where *T* is the number of leaf nodes in the tree; *w*_*j*_ represents the weights of leaf nodes; γ and λ are regularization parameters.

2. LightGBM (light gradient boosting machine)

LightGBM is a machine learning algorithm based on the gradient boosting framework, specifically designed for distributed and efficient training. Techniques, such as histogram algorithms and gradient-based one-sided sampling (GOSS; Khan et al., 2024), significantly improve training speed and efficiency. The model objective function is similar to XGBoost, but LightGBM is computationally more efficient. The histogram algorithm approximates gradient calculations by binning feature values, thereby reducing computational complexity.

3. GB (gradient boosting)

GB is an ensemble learning-based algorithm that minimizes the loss function by sequentially adding new weak learners (typically decision trees; So, 2024), where each new learner attempts to correct the residuals of the previous learner. The model prediction function is shown in Equation 3.


$$\begin{array}{l} F\left(x\right)\;=\;\sum_{m\;=\;1}^{M}{\text{h}}_{m}\left(x\right) \end{array}$$ (3)

Where *h*_*m*_(*x*) is the *m*-th weak learner (typically a decision tree).

Each iteration update is shown in Equation 4.


$$\begin{array}{l} {\text{h}}_{m}\left(x\right)\;=\;arg\underset{\text{h}}{min}\sum_{i\;=\;1}^{n}l\left[y_{i},\;F_{m1}\left(x_{i}\right)\;+\text{ h}\left(x_{i}\right)\right] \end{array}$$ (4)

Where *l* is the loss function and *F*_*m*1_(*x*) is the model prediction from the previous iteration.

4. SVM

SVM is a supervised learning algorithm for classification and regression. The radial basis function (RBF) kernel is a commonly used kernel function (Yuan et al., 2025) that maps data to a high-dimensional space to solve non-linear problems. Its decision function is shown in Equation 5.


$$\begin{array}{l} f\left(x\right)\;=\;\sum_{i\;=\;1}^{n}\alpha_{i}y_{i}K\left(x,{\;x}_{i}\right)\;+\;b \end{array}$$ (5)

Where α_*i*_ are Lagrange multipliers; *y*_*i*_ are labels, *K*(*x, x*_*i*_) is the kernel function; *b* is the bias term.

RBF kernel function is shown in Equation 6.


$$K(x,x_{i})\;=\;exp(\gamma ∥xx_{i}{∥}^{2})$$ (6)

Where γ is a parameter of the kernel function.

5. LR (logistic regression)

LR is a linear model for binary classification that maps the output of linear combinations to the (0, 1) interval through the Sigmoid function (Luo et al., 2025), representing probabilities. Its model prediction function is shown in Equation 7.


$$\begin{array}{l} P\left(y\;=\;1|x\right)\;=\;\frac{1}{1\;+\;\exp\left[\left(\beta_{0\;}+\;\beta_{1}x_{1\;}+\cdots +\beta_{p}x_{p}\right)\right]} \end{array}$$ (7)

Where β_0_, β_1_,..., β_*p*_ are model parameters.

Loss function (log-likelihood) is shown in Equation 8.


$$\begin{array}{l} L=-\sum_{i=1}^{n}[y_{i}\;log[P(y=1|x_{i}))\\ \;+(1-y_{i})log(1-P(y=1|x_{i}))] \end{array}$$ (8)

6. Linear SVC (linear support vector classifier)

Linear SVC is a linear model for classification that finds the optimal separating hyperplane by maximizing the margin (Ghosh et al., 2019). Its decision function is shown in Equation 9.


$$\begin{array}{l} f\left(x\right)\;=\;sign\left(\beta_{0\;}+\;\beta_{1}x_{1\;}+\;\cdots \;+\beta_{p}x_{p}\right) \end{array}$$ (9)

Where β_0_, β_1_,..., β_*p*_ are model parameters.

Optimization objective is shown in Equation 10.


$$\begin{array}{l} min\left(\beta 0,\beta ,\xi \right)\frac{1}{2}∥\beta {∥}^{2}+C\sum_{i\;=\;1}^{n}\xi_{i} \end{array}$$ (10)

Where *y*(β0 + β^*T*^*x*) ≥ 1ξ, ξ ≥ 0, *i* = 1, ..., *n*, *C* is the regularization parameter; ξ_*i*_ are slack variables used to handle non-separable cases.

7. Assessment indicator

Matthews correlation coefficient (MCC) is a metric used to evaluate binary classification model performance (Balayla, 2021), with the core characteristic of comprehensively considering all four types of samples in the confusion matrix [true positives (TP), true negatives (TN), false positives (FP), false negatives (FN)]. Its results range from −1 to 1, where 1 indicates perfect prediction or complete matching, −1 indicates complete inconsistency between predictions and actual values, and 0 indicates performance no better than random prediction. The MCC formula is shown in Equation 11.


$$\begin{array}{l} MCC=\frac{TP\;\times \;TN\;-FP\;\times \;FN}{\sqrt{\begin{array}{c} \left(TP\;+\;FP\right)\;\left(TP\;+\;FN\right)\;\left(TN\;+\;FP\right)\\ \left(TN\;+\;FN\right) \end{array}}} \end{array}$$ (11)

## 4 Results and discussions

### 4.1 Workload differences in left and right steep turns

To quantitatively assess differences in workload between left and right turns, the NASA Task Load Index (NASA-TLX) was used to assess pilots' subjective MWL. The NASA-TLX is a multidimensional rating scale widely validated for workload assessment in aviation and human-machine systems (Hart and Staveland, 1988). It evaluates subjective workload through six dimensions: mental demand, physical demand, temporal demand, performance, effort, and frustration. Each subscale was rated on a 0~10 score (i.e., 21 discrete scale points from 0 to 10). Dimension weights were derived from the NASA-TLX pairwise comparison procedure and normalized. The overall TLX score was computed as the sum of the six weighted subscale scores (range: 0~10).

**Figure 3:**
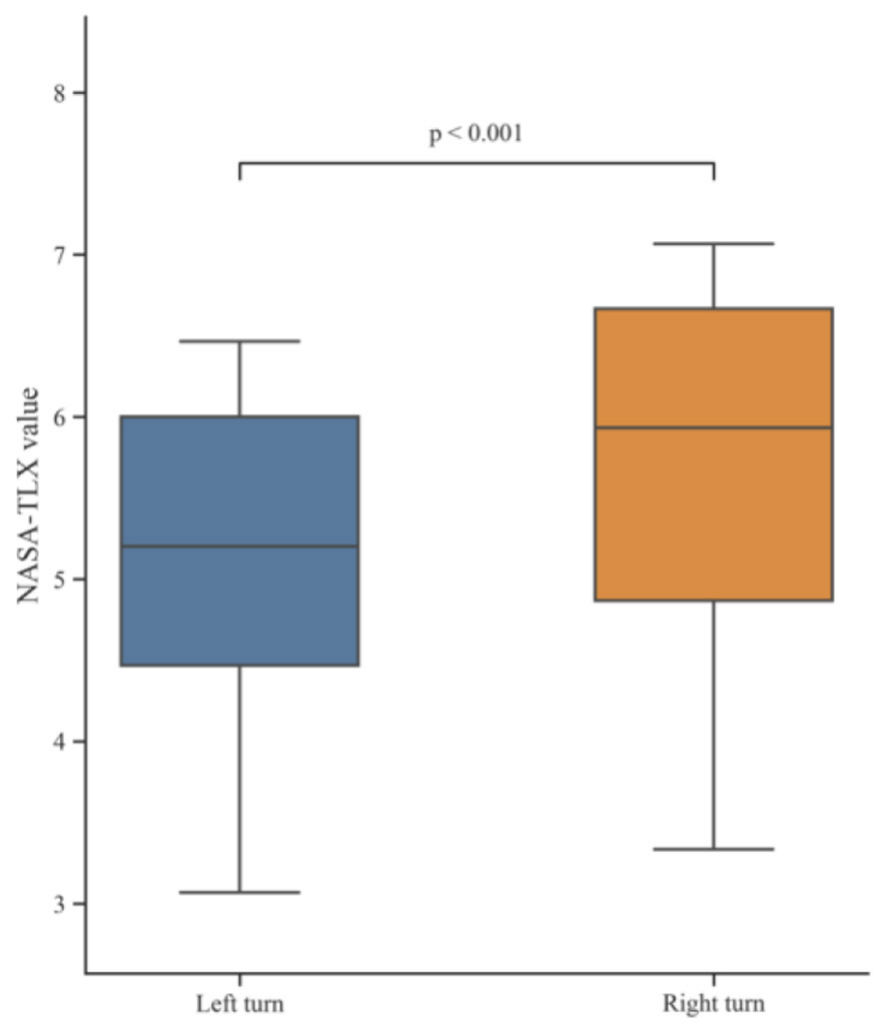
Statistical analysis results of NASA-TLX scores.

Given the within-subject experimental design, a paired-sample *t*-test was conducted to compare NASA-TLX total scores between the left and right steep-turn conditions. Normality of the difference scores was verified using the Shapiro–Wilk test (*p* > 0.05). The analysis revealed a statistically significant difference in workload between the two maneuvers (*p* < 0.001, Cohen's *d* = 0.52), indicating a medium effect size. As shown in Figure 3, the mean NASA-TLX total score for left steep turns was 4.98 ± 1.06, whereas right steep turns yielded a significantly higher mean score of 5.55 ± 1.13. This indicates a higher increase in perceived workload for right turns than for left turns.

Although this study confirmed that MWL differences are the primary driver of EEG feature disparities between left and right steep turns, other potential confounding factors cannot be entirely excluded. To clarify the sources of overall workload asymmetry and minimize the possibility that these effects are driven exclusively by non-cognitive confounders, focused subscale analyses were conducted on the mental demand and physical demand dimensions of the NASA-TLX, which directly correspond to cognitive resource requirements and control-force-related exertion, respectively. Results showed significantly higher mental demand during right turns compared to left turns (left: 1.328 ± 0.737, right: 1.428 ± 0.528, paired *t*-test: *p* = 0.047 <0.05), whereas physical demand showed no significant difference between conditions (left: 1.106 ± 0.931, right: 1.056 ± 1.147, paired *t*-test: *p* = 0.70 > 0.05). This pattern indicates that the workload asymmetry between left and right turns aligns more closely with increased cognitive demands (e.g., state monitoring and instrument cross-checking) rather than purely physical exertion. The observed right-left workload difference may arise from (i) aircraft asymmetric propulsion effects that alter control requirements, (ii) procedural familiarity and skill automation, and (iii) asymmetric visual references in a left-seat cockpit.

Single-engine propeller aircraft exhibit asymmetric thrust and torque effects that may differentially impact control force requirements between left and right turns. During left steep turns, these effects generate a natural yawing tendency that partially counteracts adverse yaw from aileron deflection (Federal Aviation Administration, 2022), reducing the magnitude of rudder input required for coordinated flight and decreasing motor coordination demands. Conversely, right steep turns require pilots to actively counteract these propulsive effects, necessitating greater and more sustained rudder deflections to maintain coordination (Diehl, 1991). This increased control activity elevates both precision demands and continuous monitoring requirements.

Standard traffic patterns, holding procedures, and most published instrument approach procedures predominantly employ left turns, resulting in pilots accumulating substantial training and operational experience with left-turn execution. This extensive practice promotes greater automaticity of associated motor skills, thereby reducing cognitive resource demands during task execution (Casner et al., 2014). Conversely, the relative unfamiliarity of right-steep turns may require pilots to allocate more conscious attention to control inputs and performance monitoring, thus increasing mental demand.

The left-seat cockpit configuration enables pilots to maintain direct visual contact with the turn radius during left turns, providing convenient external visual cues for estimating bank angle and turn performance assessment (Gibb et al., 2011). However, during right steep turns, the aircraft structure obstructs much of the pilot's natural sightline toward the turning direction, reducing the availability of external visual references. This visual asymmetry compels greater reliance on instrument cross-checking and increases the cognitive workload associated with maintaining spatial situation awareness (Dehais et al., 2014). This shift from predominantly external to internal attentional focus may explain the elevated mental demand observed during right-turn conditions.

In summary, the combined effects of aerodynamic factors, procedural familiarity differences, and visual reference asymmetry create cumulative workload demands during right steep turns. Pilots must simultaneously manage increased control forces, compensate for reduced motor automaticity, process greater volumes of instrument information, and maintain aircraft state within narrow performance tolerances. These multidimensional demands on cognitive, physical, and perceptual resources ultimately manifest as statistically significant elevations in NASA-TLX scores. They may amplify MWL by increasing cognitive resource requirements. Furthermore, the finding that right-turn tasks impose a higher workload aligns closely with the widely recognized operational experience in aviation that “left turns are easier to execute,” further validating the ecological validity of the experimental results.

### 4.2 Model prediction

**Figure 4:**
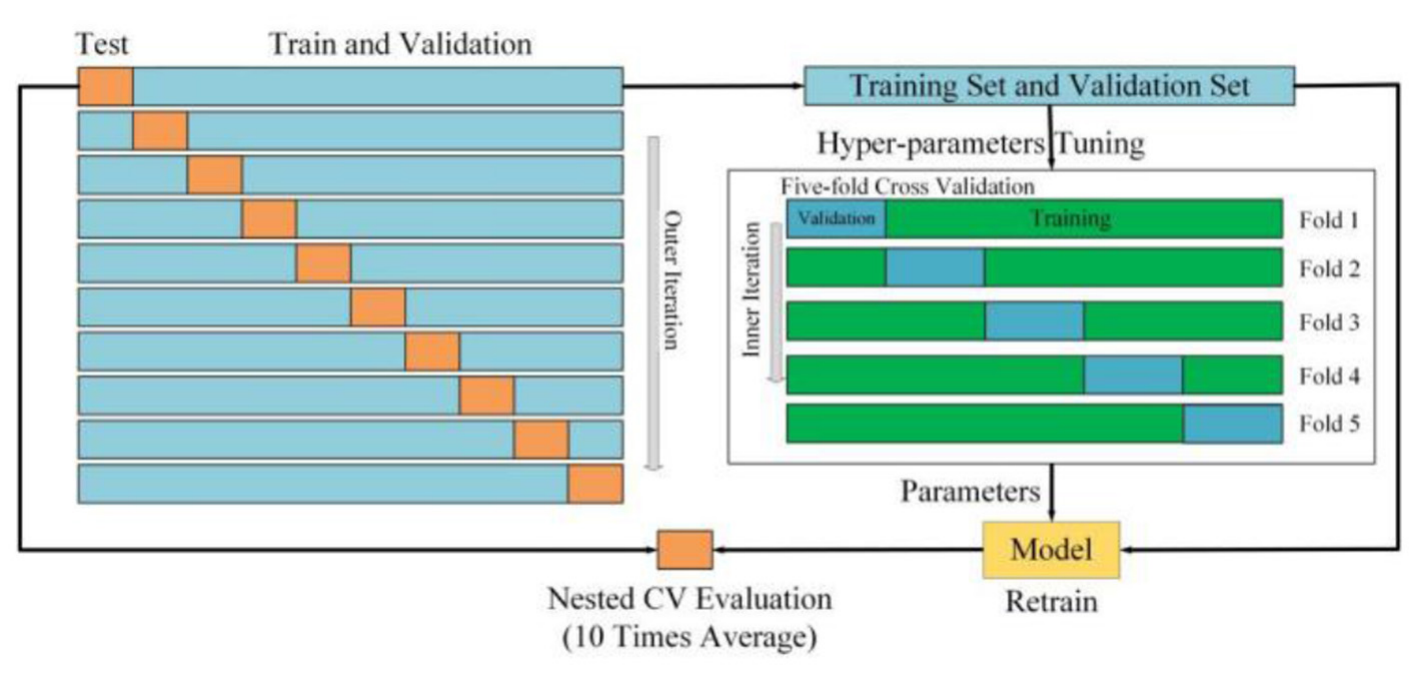
NCV framework ensuring unbiased model optimization and robust performance evaluation.

To rigorously assess and compare the generalization performance of multiple machine learning classifiers, an automated experimental framework based on nested cross-validation (NCV) was designed and implemented, as shown in Figure 4. The framework ensures an unbiased estimate of model performance by strictly separating hyperparameter optimization, feature selection, and final performance evaluation. The dataset used in this study was collected from 37 pilots performing left and right steep turns, with EEG features obtained during the task. The raw data were preprocessed to generate a binary classification dataset, with missing values imputed using mean imputation and standardized within a scikit-learn pipeline to ensure consistency and prevent data leakage during cross-validation. Preprocessing steps were applied independently to the training data in each cross-validation fold, thereby avoiding any risk of information leakage.

The nested cross-validation protocol consists of two nested loops: the outer loop uses 10-fold stratified cross-validation (StratifiedGroupKFold) for performance evaluation, while the inner loop employs 5-fold stratified cross-validation for feature selection and hyperparameter tuning. The inner loop incorporates unsupervised feature ranking, feature subset exploration, and hyperparameter grid search. In each inner fold, feature subsets and hyperparameters were optimized simultaneously on the training data, with the best feature ratio and hyperparameter configuration selected via majority voting. In the outer loop, the optimized model configuration was used to train on all the training data, and the performance was evaluated on the independent test set. For unbiased performance evaluation, the entire procedure was repeated 10 times with different random seeds (rng = 42 + repeat times), and the performance indices were calculated as the mean ± standard deviation. Our nested cross-validation pipeline used variance-based ranking for feature preselection. To improve interpretability, we additionally reported RF-based feature importance as a *post-hoc* model explanation, and supplemented it with variance-top feature statistics to address concerns about model dependence.

The 800 EEG features were ranked in descending order by variance. Subsequently, features were selected at five percentages (20%, 40%, 60%, 80%, and 100% of the ranked features) to serve as inputs to the six classifiers. The results, presented in Table 2, represent the averaged outcomes from the 10-time repeated nested cross-validation. It was found that the XGBoost, LightGBM, and GB models exhibit high robustness to varying feature selection ratios, effectively adapting to changes in the number of features while achieving excellent performance across multiple evaluation metrics. Conversely, LR, Linear SVC, and SVM perform poorly at high feature ratios, with a particularly noticeable decline in accuracy and precision, indicating that continuing to add redundant or weakly correlated features does not yield further gains (Fernández-Delgado et al., 2014) and may even introduce noise, thereby weakening model generalization capability.

**Table 2:** Mean classifier results (mean ± std) under different feature selection percentages.

Feature percentage	Model	Accuracy	Precision	Recall	F1	ROC-AUC	MCC
20%	LR	0.8686 ± 0.0471	0.8745 ± 0.0652	0.8713 ± 0.0856	0.8686 ± 0.0506	0.9312 ± 0.0568	0.8755 ± 0.0470
	Linear SVC	0.8657 ± 0.0467	0.8713 ± 0.0652	0.8690 ± 0.0861	0.8658 ± 0.0505	0.9287 ± 0.0562	0.8745 ± 0.0466
SVM (RBF Kernel)	0.8394 ± 0.0555	0.8155 ± 0.0778	0.8962 ± 0.0730	0.8496 ± 0.0481	0.9357 ± 0.0414	0.8391 ± 0.0558
GB	0.8999 ± 0.0279	0.9080 ± 0.0439	0.8963 ± 0.0712	0.8991 ± 0.0321	0.9676 ± 0.0183	0.9000 ± 0.0277
XGBoost	0.8992 ± 0.0275	0.9049 ± 0.0436	0.8986 ± 0.0699	0.8988 ± 0.0314	0.9685 ± 0.0173	0.8993 ± 0.0273
LightGBM	0.9018 ± 0.0275	0.9073 ± 0.0437	0.9001 ± 0.0702	0.9008 ± 0.0317	0.9715 ± 0.0152	0.9014 ± 0.0274
40%	LR	0.8654 ± 0.0476	0.8695 ± 0.0625	0.8699 ± 0.0844	0.8656 ± 0.0516	0.9164 ± 0.0646	0.8654 ± 0.0475
	Linear SVC	0.8644 ± 0.0466	0.8680 ± 0.0616	0.8693 ± 0.0834	0.8648 ± 0.0492	0.9135 ± 0.0651	0.8644 ± 0.0465
SVM (RBF Kernel)	0.8339 ± 0.0536	0.8012 ± 0.0736	0.9069 ± 0.0660	0.8469 ± 0.0453	0.9300 ± 0.0415	0.8334 ± 0.0539
GB	0.8992 ± 0.0284	0.9066 ± 0.0451	0.8967 ± 0.0707	0.8986 ± 0.0326	0.9670 ± 0.0186	0.8993 ± 0.0282
XGBoost	0.8996 ± 0.0273	0.9063 ± 0.0444	0.8978 ± 0.0694	0.8991 ± 0.0311	0.9684 ± 0.0169	0.8997 ± 0.0271
LightGBM	0.9013 ± 0.0271	0.9086 ± 0.0437	0.8997 ± 0.0695	0.9012 ± 0.0311	0.9713 ± 0.0153	0.9018 ± 0.0269
60%	LR	0.8509 ± 0.0491	0.8495 ± 0.0643	0.8640 ± 0.0844	0.8526 ± 0.0519	0.9067 ± 0.0649	0.8508 ± 0.0491
	Linear SVC	0.8474 ± 0.0485	0.8460 ± 0.0646	0.8609 ± 0.0824	0.8495 ± 0.0499	0.9012 ± 0.0664	0.8474 ± 0.0485
SVM (RBF Kernel)	0.8087 ± 0.0552	0.7710 ± 0.0725	0.8995 ± 0.0672	0.8265 ± 0.0451	0.9122 ± 0.0467	0.8081 ± 0.0556
GB	0.8992 ± 0.0275	0.9052 ± 0.0450	0.8984 ± 0.0693	0.8988 ± 0.0313	0.9669 ± 0.0186	0.8993 ± 0.0273
XGBoost	0.8980 ± 0.0278	0.9056 ± 0.0447	0.8952 ± 0.0704	0.8974 ± 0.0318	0.9686 ± 0.0169	0.8981 ± 0.0276
LightGBM	0.9000 ± 0.0275	0.9078 ± 0.0448	0.8969 ± 0.0697	0.8993 ± 0.0314	0.9715 ± 0.0147	0.9001 ± 0.0273
80%	LR	0.8454 ± 0.0503	0.8443 ± 0.0660	0.8597 ± 0.0924	0.8470 ± 0.0561	0.9016 ± 0.0649	0.8454 ± 0.0502
	Linear SVC	0.8424 ± 0.0505	0.8402 ± 0.0668	0.8582 ± 0.0865	0.8448 ± 0.0531	0.8954 ± 0.0666	0.8424 ± 0.0505
SVM (RBF Kernel)	0.7997 ± 0.0547	0.7654 ± 0.0720	0.8865 ± 0.0722	0.8174 ± 0.0454	0.9041 ± 0.0470	0.7992 ± 0.0550
GB	0.8988 ± 0.0275	0.9058 ± 0.0447	0.8967 ± 0.0695	0.8982 ± 0.0315	0.9665 ± 0.0185	0.8989 ± 0.0273
XGBoost	0.8978 ± 0.0276	0.9057 ± 0.0441	0.8944 ± 0.0708	0.8970 ± 0.0319	0.9683 ± 0.0165	0.8979 ± 0.0275
LightGBM	0.9010 ± 0.0275	0.9087 ± 0.0442	0.8980 ± 0.0694	0.9004 ± 0.0314	0.9713 ± 0.0147	0.9011 ± 0.0274
100%	LR	0.8374 ± 0.0481	0.8333 ± 0.0637	0.8556 ± 0.0828	0.8402 ± 0.0504	0.8964 ± 0.0630	0.8373 ± 0.0481
	Linear SVC	0.8343 ± 0.0487	0.8293 ± 0.0657	0.8544 ± 0.0796	0.8378 ± 0.0490	0.8917 ± 0.0652	0.8342 ± 0.0488
SVM (RBF Kernel)	0.7893 ± 0.0539	0.7544 ± 0.0700	0.8804 ± 0.0729	0.8085 ± 0.0445	0.8951 ± 0.0478	0.7888 ± 0.0542
GB	0.8991 ± 0.0276	0.9077 ± 0.0442	0.8951 ± 0.0708	0.8983 ± 0.0319	0.9662 ± 0.0183	0.8992 ± 0.0274
XGBoost	0.8987 ± 0.0279	0.9076 ± 0.0434	0.8941 ± 0.0713	0.8978 ± 0.0322	0.9683 ± 0.0166	0.8988 ± 0.0277
LightGBM	0.9011 ± 0.0279	0.9100 ± 0.0441	0.8965 ± 0.0706	0.9002 ± 0.0323	0.9715 ± 0.0149	0.9012 ± 0.0277

Based on F1 score performance, the top three models are LightGBM, XGBoost, and GB. LightGBM employs a histogram-based algorithm to discretize features, thereby reducing computational complexity (Ke et al., 2017). Across different feature percentages, its F1 score consistently remains around 0.90, demonstrating a concentrated distribution and leading performance while preserving decision boundary accuracy. XGBoost's regularized objective function effectively mitigates overfitting (Nalluri et al., 2020), exhibiting minimal F1 score fluctuations across the entire range of feature percentages, which reflects exceptional stability. GB maintains F1 scores above 0.898 throughout, positioning only marginally below the top two performers. Conversely, LR, Linear SVC, and SVM exhibit significantly lower F1 scores, indicating heightened sensitivity to variations in feature percentage and data distributions, with greater performance volatility characteristics intrinsic to the limitations of linear assumptions. Linear models rely on the linear separability of features (Hsh, 2002). As the feature percentage increases from 20% to 100%, effective information is diluted by redundant features, impairing the models' ability to capture complex patterns and leading to a sustained decline in F1 scores.

**Figure 5:**
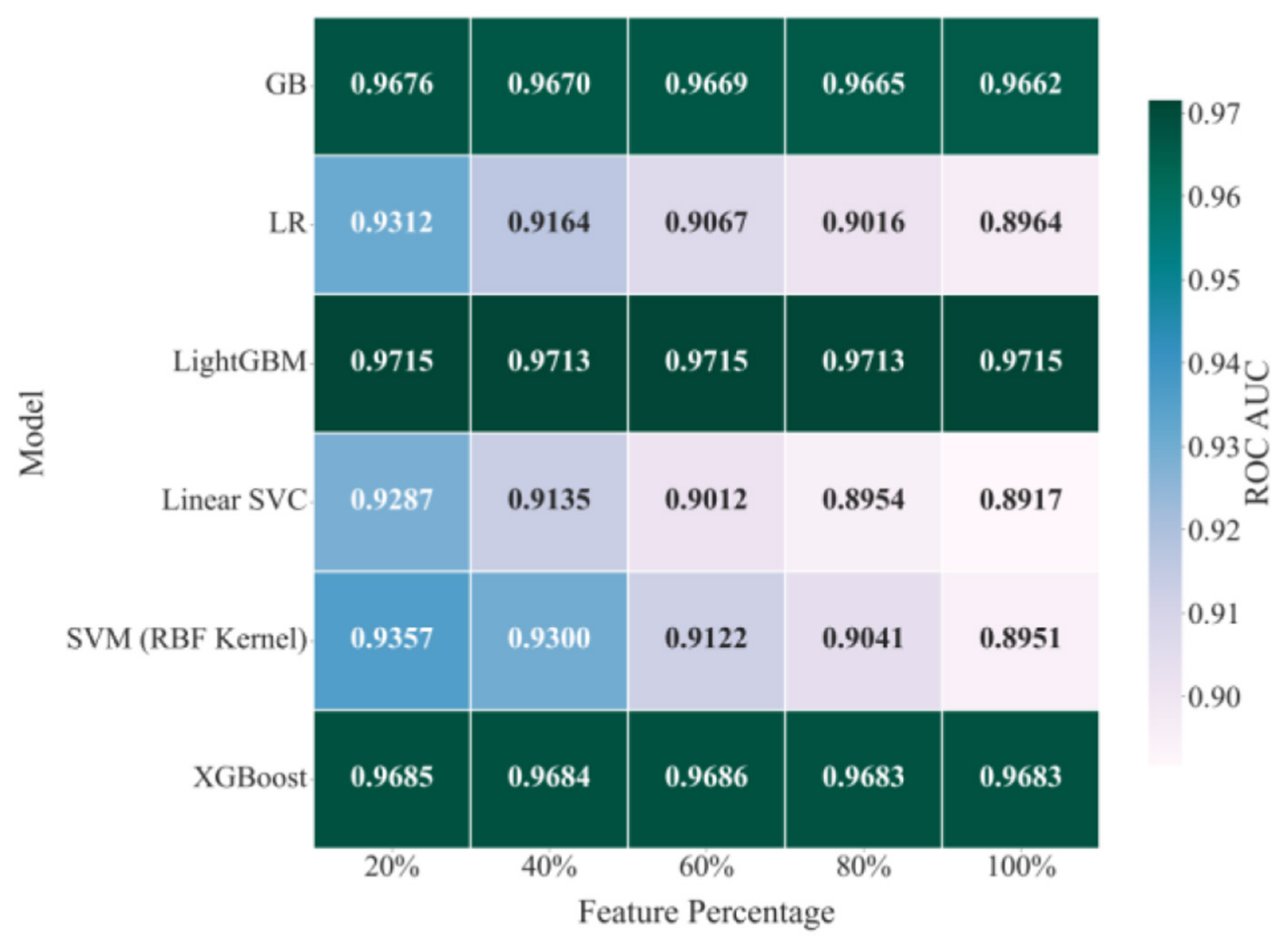
Heatmap of ROC AUC for different feature percentages and classifiers.

Figure 5 presents a heatmap of ROC AUC corresponding to different feature percentages and classifiers. It was found that GB tree-based models (LightGBM, XGBoost, and GB) consistently maintain high and stable ROC AUC scores throughout the entire feature percentage range, demonstrating excellent predictive performance and robustness, and a trend of reaching optimality at low feature percentage (Pinheiro et al., 2025) and stable maintenance at a high percentage. Among all evaluated models, LightGBM demonstrates superior ROC AUC performance, consistently maintaining elevated values across the entire spectrum of feature percentages, followed by XGBoost, while GB stabilizes at 0.9662 to 0.9676. LightGBM's ROC AUC significantly outperforms linear models and SVM and surpasses those of GB and XGBoost. By leveraging histogram-based algorithms and leaf-wise splitting strategies, LightGBM enhances training efficiency while achieving marginally higher accuracy than XGBoost across all feature percentages, substantially exceeding the performance of linear models and SVM. This superiority reflects the universal advantages of the GB framework (Ke et al., 2017). XGBoost optimizes its objective function through second-order Taylor expansion and incorporates tree complexity regularization terms. This approach effectively captures non-linear feature interactions while mitigating overfitting, thereby enabling consistent and robust predictive performance across varying feature scales (Chen and Guestrin, 2016).

Linear models (LR, Linear SVC) and SVM models exhibit lower ROC AUC scores compared to GB tree-based models. Moreover, as the feature percentage increases from 20% to 100%, their ROC AUC scores show a noticeable decreasing trend. Linear models are sensitive to redundant and collinear features. As the feature percentage increases (introducing more non-essential or redundant features), multicollinearity leads to unstable coefficient estimates, which in turn reduce the model's generalization ability. In the case of SVM, if a linear kernel is used, it behaves similarly to linear models and is prone to the influence of high-dimensional redundant features. When a non-linear kernel is employed, the computational complexity of the kernel function increases significantly as the feature scale expands, making the model more susceptible to overfitting, particularly when the percentage of effective features is diluted. Consequently, the ROC AUC of SVM decreases as the feature percentage increases.

From the perspective of the underlying mechanisms driving differences in model performance, the superiority of gradient boosting tree models stems primarily from their capacity to accommodate the non-linear characteristics of EEG signals. As a direct reflection of neural activity in the brain, the relationship between EEG features and workload states is inherently non-linear rather than simply linear. The interactive effects among features such as power in different frequency bands and entropy values, along with their time-varying properties, exhibit complex non-linear patterns. LightGBM, XGBoost, and similar models can effectively capture these non-linear associations by integrating multiple weak classifiers, which constitutes the core reason for their stable performance across different feature proportions. Conversely, the performance degradation observed in linear models and SVM reflects the inadequacy of linear assumptions or single-kernel mappings in adapting to the high-dimensional non-linear nature of EEG data.

Moreover, the finding in this study that model performance is optimal at 20% feature proportion holds significant practical implications. This indicates that the critical information in EEG signals related to workload differences between left and right steep turns is highly concentrated in a small subset of high-variance features, including redundant features, which interfere with the model's recognition of core patterns. This result aligns with the neuroscience perspective that neural representations of specific cognitive tasks exhibit sparsity. When the brain executes complex flight tasks such as steep turns, only specific neural activity patterns in particular brain regions (such as the prefrontal cortex and motor cortex), including theta/gamma band oscillations and changes in non-linear complexity, are highly relevant to task demands. By selecting high-variance features, the model precisely captures these core neural representations. From an engineering application perspective, this finding provides crucial support for developing lightweight pilot workload-monitoring systems: high-precision workload classification can be achieved by extracting only a small set of key EEG features, substantially reducing computational burden on devices and data transmission requirements.

**Figure 6:**
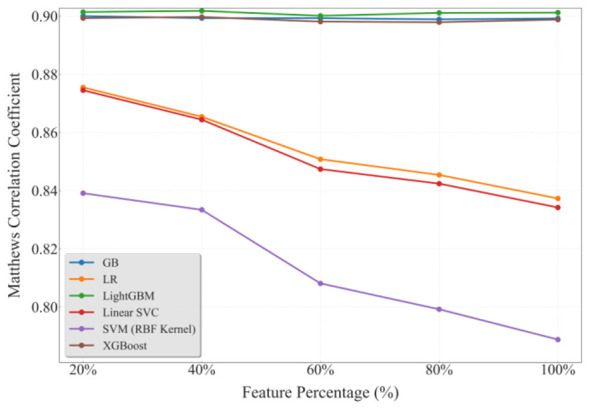
Line plots of MCC for different feature percentages and classifiers.

Figure 6 demonstrates the MCC variations of six machine learning models at different feature percentages. The MCC values of the gradient boosting tree models (GB, XGBoost, and LightGBM) remain consistently high, with minimal fluctuation across the entire feature percentage range, demonstrating stable and excellent predictive performance and robustness. Specifically, LightGBM exhibits optimal MCC performance, peaking at 40% feature percentage. GB and XGBoost demonstrate comparable performance, with all three models significantly outperforming linear models (LR, Linear SVC) and SVM. For XGBoost, MCC reaches its maximum at 20% feature percentage before gradually declining, reflecting the model's strong ability to extract critical features within low-dimensional spaces. Through random feature subset sampling during training, XGBoost enhances adaptability to sparse feature scenarios, thereby effectively capturing essential information even at 20% feature percentage.

Conversely, linear models (Linear SVC, LR) exhibit a persistent decline in MCC values as feature percentage increases. At a low feature percentage (20%), both LR and Linear SVC achieve their optimal MCC performance across the entire feature percentage range; when feature percentage increases to 100%, MCC values for both models decrease substantially, consistently remaining inferior to those of tree-based models. This phenomenon stems from linear models' reliance on linear combinations of features. Under low feature percentage conditions, insufficient informative content (Lange et al., 2019) prevents these models from capturing complex patterns (e.g., non-linear decision boundaries), thereby limiting their performance ceiling. Even with increased feature quantity, the inherent linear relationship constraints fail to adequately fit the non-linear nature of real-world data, while redundant features introduce noise that further degrades model stability, ultimately leading to continuous MCC deterioration.

For the SVM (RBF Kernel), MCC values show a pronounced decline as feature percentage increases, indicating extreme sensitivity to variations in feature percentage. At 20% feature percentage, SVM achieves its optimal MCC across the entire interval; when feature percentage increases to 100%, MCC decreases substantially, with overall performance inferior to both tree-based and linear models. Although the RBF kernel employed by SVM can address non-linear problems through high-dimensional mapping, feature percentage directly affects the kernel matrix sparsity and computational efficiency. Under low feature percentage conditions, kernel mapping fails to capture sufficient non-linear feature interactions, resulting in moderate MCC values. As the feature percentage increases, redundant information exacerbates the complexity of kernel mapping, inducing overfitting and ultimately leading to a significant degradation in MCC.

It is important to emphasize that the MCC, as a comprehensive evaluation metric that simultaneously considers all four categories of samples in the confusion matrix, provides a more reliable reflection of model performance in practical applications through its variation trends. The consistently high MCC values achieved by gradient boosting tree models across the full range of feature proportions demonstrate their robust stability in recognizing both positive and negative samples (left-turn/right-turn tasks), with minimal susceptibility to class imbalance or extreme samples. This characteristic is critical for real-time pilot workload monitoring, as misclassification of either task category could pose flight safety risks. The MCC degradation observed in linear models and SVM, particularly the performance deterioration at high feature proportions, further validates the applicability and superiority of gradient boosting tree models for EEG signal classification tasks. Additionally, the performance differences among models observed in this study provide valuable direction for future research. Subsequent investigations could integrate the feature selection mechanisms of gradient boosting tree models with the end-to-end modeling capabilities of deep learning approaches to further enhance the accuracy and generalizability of workload classification.

**Figure 7:**
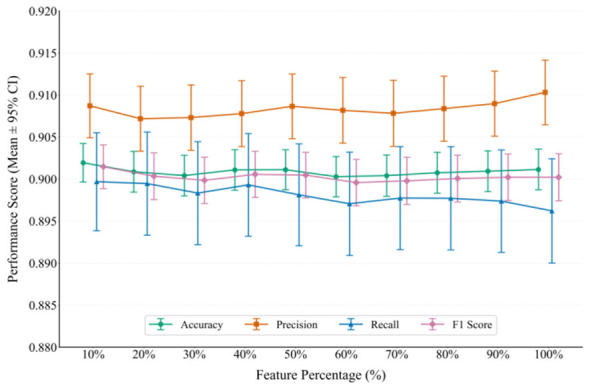
Classification performance of the LightGBM model under different feature percentages.

To further investigate the optimal feature ratio, additional targeted tests were conducted on the best-performing gradient boosting model (LightGBM) using feature percentages of 10%, 30%, 50%, 70%, and 90%. Figure 7 presents the mean values and 95% confidence intervals (error bars) of four core classification metrics (accuracy, precision, recall, and F1 score) for the LightGBM model across a continuous range of feature ratios from 10% to 100% at 10% increments. The results demonstrate that all performance metrics remained stable throughout the systematic evaluation with 10% step increments, exhibiting neither significant upward nor downward trends as the feature ratio increased. The mean values of all metrics were concentrated within a narrow range, and the 95% confidence intervals showed substantial overlap, indicating no statistically significant differences in performance across feature ratios. Notably, even at the minimal feature ratio of 10%, model performance approached levels achieved at higher percentages, confirming both the effectiveness of the selected feature subsets and the model's robustness to feature dimensionality reduction. Importantly, rather than testing only limited discrete feature ratios, a comprehensive continuous search was performed at 10% intervals across the full range, combined with hyperparameter optimization and majority voting strategies within a nested cross-validation framework, ensuring stability and generalizability while eliminating the risk of overlooking superior feature percentages.

Although performance differences across feature ratios were small, 10% provided the most parsimonious representation while maintaining the best classification performance compared to other ratios, except for precision. Based on the above results, we adopted a 10% feature ratio as the optimal feature proportion. From a model-selection perspective, this choice balances predictive accuracy and feature compactness, reducing the risk of interpretation bias introduced by redundant variables. Therefore, the subsequent feature-importance uses the top-ranked 10% subset as well.

### 4.3 Features statistical analysis for left/right steep turns

To systematically identify EEG features that distinguish left and right steep turns, a two-step integrated analysis framework was designed to combine statistical rigor and functional relevance. First, statistical analysis was conducted to screen features that showed significant differences between the two turn conditions, ensuring the identified markers were not randomly distributed. Second, feature importance analysis was performed to verify which statistically significant features actually drove the classification of left/right turns, avoiding over-reliance on statistical significance without practical predictive value. This integrated approach addresses the limitations of single-step analysis and enhances the reliability of discriminative feature identification.

#### 4.3.1 Significant EEG features analysis

Due to the sample size being less than 50, the Shapiro–Wilk test (Jatupornpoonsub et al., 2021) was used to assess the normality of EEG feature data with a significance level of α = 0.05 as the threshold. If the *p*-value > 0.05, the feature was considered to follow a normal distribution in the corresponding group; otherwise, it was classified as non-normally distributed. For significance testing, if both groups followed normal distributions, an independent samples *t*-test was used; if at least one group was non-normal, the Mann–Whitney *U* non-parametric test was used. With α = 0.05 as the threshold, if the *p*-value <0.05, the feature was considered statistically significant, resulting in a total of 250 significant EEG features.

To control for inflated false positives due to multiple testing across 250 EEG features, the Benjamini–Hochberg false discovery rate (FDR) correction was applied (Benjamini and Hochberg, 1995). Statistical significance was defined at q <0.05, where q denotes the FDR-adjusted *p*-value. While *p*-values reflect the probability of observing the data under the null hypothesis for a single test, *q*-values indicate the expected percentage of false positives among the set of results deemed significant. Following correction, 132 significant features remained (reduced from 250 before correction) and were subsequently used in the downstream analyses.

**Figure 8:**
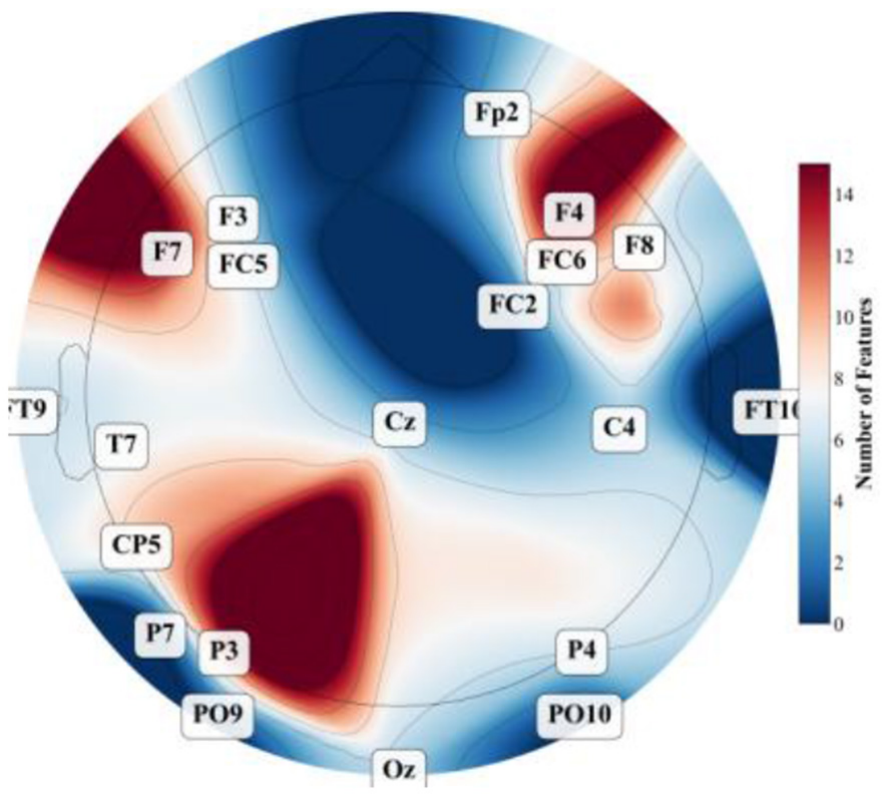
Scalp topographic map of significant features.

The scalp topographic map (as shown in Figure 8) is employed to visualize the spatial distribution of the number of statistically significant EEG features, arranged according to the International 10–20 System (a standardized framework for EEG electrode placement). The color gradient (transitioning from blue cool tones to red warm tones) intuitively reflects the relative abundance of feature counts at each electrode site, with the color bar serving as a legend that clearly indicates the range of feature numbers: from 0 (blue, lowest abundance) to 18 (red, highest abundance). The topographic map reveals a non-uniform spatial distribution of feature counts across the scalp: regions with warm tones exhibit higher feature counts, suggesting that these brain areas may demonstrate enhanced EEG activity during specific cognitive tasks or states; conversely, regions with cool tones show lower feature counts, more likely corresponding to relatively inactive brain areas or regions not directly involved in the target cognitive processes.

The spatial distribution of significant features is highly consistent with brain functional parcellation theory. The warm-colored regions, including the prefrontal cortex (F4, F7) and frontocentral areas (FC6), correspond precisely to the core brain regions responsible for executing complex motor control and cognitive coordination tasks. This alignment is consistent with the demands of steep turn tasks, where pilots must precisely maintain bank angles, coordinate multiple control surfaces, and integrate instrument and visual information. From a neuroanatomical perspective, the prefrontal cortex is responsible for motor intention encoding, decision-making, and multimodal information integration, whereas the frontocentral region serves as a sensorimotor interface involved in sensorimotor transformation. The enrichment of significant features in these brain regions reflects the selective activation of decision-making, control, and functional integration networks during steep turn tasks. Conversely, cold-colored regions such as the occipital lobe and posterior parietal areas exhibit fewer significant features, likely because these brain regions primarily subserve basic visual processing and somatosensory integration, which are not core functional components during steep turn tasks. This distribution pattern further validates the effectiveness and functional relevance of the feature selection approach employed in this study.

#### 4.3.2 Important EEG features analysis

In the nested cross-validation framework, feature ranking within each inner fold was based on variance calculated from the corresponding training set, with features selected in descending order of variance. However, due to inherent distributional differences across training sets across folds, feature rankings between folds lacked direct comparability. Therefore, after evaluating model performance, a unified feature importance analysis was conducted as a *post-hoc* interpretability step. Following an initial statistical significance-based filtering of the full feature set, a Random Forest model was trained on the full dataset using only the retained significant features to assess the global importance of core features for classification. The algorithm was configured with 100 decision trees and a random seed of 42 to ensure reproducibility. Feature importance was quantified by the mean decrease in Gini impurity (Gemayel et al., 2024), with all features ranked in descending order. Ranking stability was validated through 1,000 bootstrap resampling iterations.

**Figure 9:**
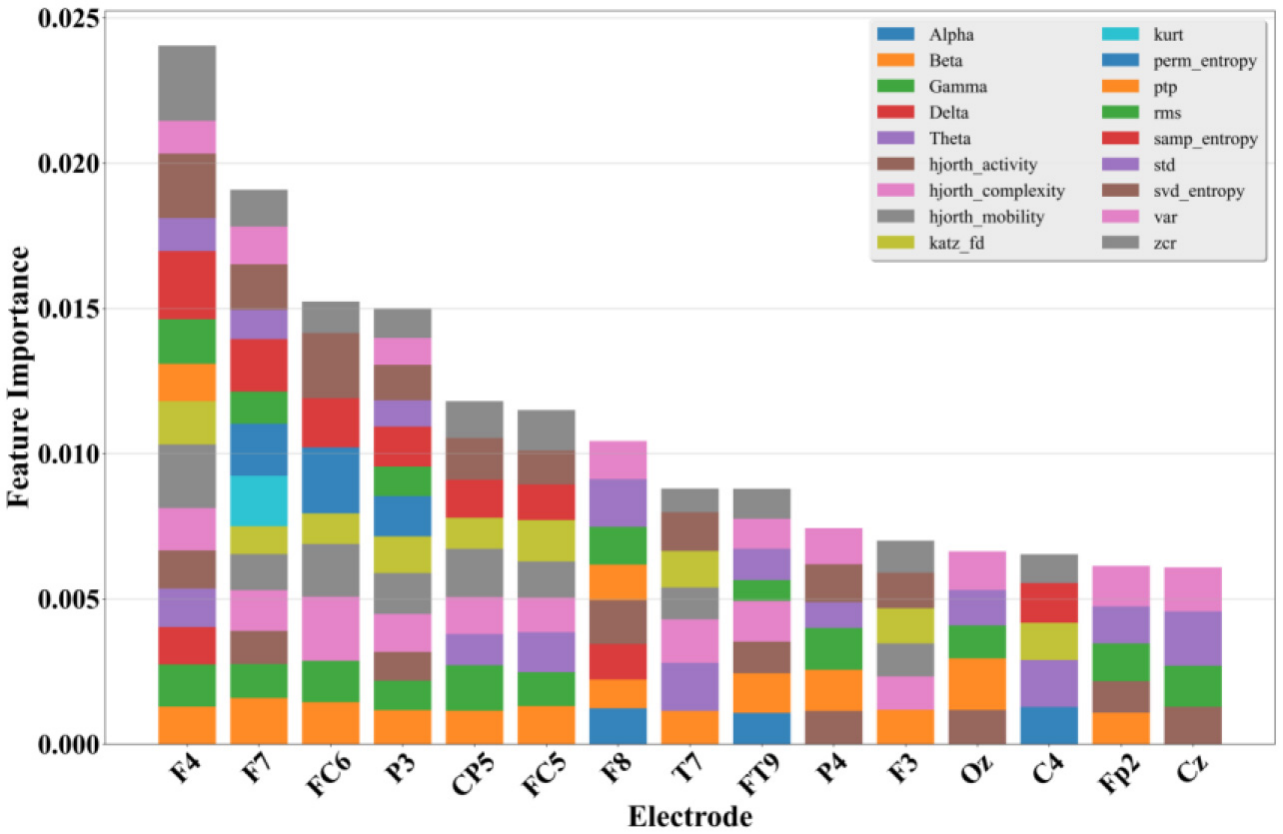
Important features sorted by electrode and Random Forest.

Figure 9 presents a stacked bar chart of 124 of the total 132 important EEG features in the top-15 EEG electrodes (primarily frontal regions such as F4, F7, FC6, etc.) and multiple feature types (frequency band power: Alpha, Beta, etc.; complexity indices: hurst_complexity, perm_entropy, etc.) during steep slope turning tasks. It was found that features from frontal-associated electrodes (F4, F7, and FC6) exhibited significantly higher importance than those from temporal (T7) and parietal (C6) regions, with the F4 electrode showing the most prominent peak in feature importance, reflecting that the core neural activity during steep slope turning is concentrated in the frontal region. Subjects need to precisely maintain slope angle, coordinate multiple control surfaces, and integrate instrument and visual information—these goal-directed control demands heavily rely on frontal lobe decision-making and regulatory functions (Miller and Cohen, 2001). The frontal lobe encodes motor intentions for target slope angles, drives motor cortex activation, and simultaneously integrates multimodal perceptual information, thus explaining the predominant importance of frontal electrodes in feature extraction. Notably, among the 132 FDR-retained significant features, 124 (93.94%) were distributed within the top 15 electrodes shown in Figure 9, whereas only eight features (6.06%) were located outside these electrodes; therefore, this top-15 electrode visualization captures the vast majority of the significant feature structure.

Furthermore, frequency band power (Alpha/Beta/Gamma) accounts for the highest percentage at core frontal electrodes (F4, F7), mapping the contribution of local neural oscillations. Complexity indices are more prominent at FC6 (middle frontal gyrus), Fz (frontal midline), and other electrodes, suggesting regional integration of non-linear neural dynamics. Attitude stabilization during steep-slope turning is accompanied by oscillatory dynamics of local neural clusters. When the motor cortex is activated, Alpha power often decreases, reflecting attention focus toward the task; in Beta/Gamma frequency bands, Beta oscillations are associated with motor preparation (Plank, 2009), while Gamma synchronization marks local neuronal cooperation.

Steep turns require multi-surface coordination and monitoring of multiple sources of information, leading to increased cognitive load and non-linear neural activity dynamics. These indices reflect the efficiency of cross-regional information integration, with a higher percentage of complexity features in the frontal lobe (decision-making) and frontal-central region (FC6, motor-cognitive interface), corresponding to the functional division between motor control and cognitive coordination.

From a neurodynamic perspective, the predominance of feature importance in prefrontal regions reflects the core demands for cognitive-motor integration during steep-turn tasks. The reduction in Alpha-band power is directly associated with attentional resource allocation, beta-band oscillations participate in motor program preparation and execution, and Gamma-band synchronization supports local neuronal cooperation during fine motor control. The power changes across these frequency bands collectively constitute the neural oscillatory foundation for implementing motor intention. Furthermore, the high importance of complexity metrics such as permutation entropy and singular value decomposition entropy indicates the non-linear dynamical characteristics of neural activity in the brain during steep-turn tasks. Information exchange among multiple brain regions does not simply follow linear superposition but rather exhibits complex dynamic coupling patterns, and differences in these patterns represent the key neural signatures distinguishing left from right steep turn tasks. Furthermore, the F4 electrode, as a core site of the left prefrontal cortex, exhibits peak feature importance consistent with hemispheric lateralization theory. The left prefrontal cortex plays a dominant role in complex motor decision-making and multi-objective coordination, which aligns with findings of elevated high-frequency energy and complexity in the left hemisphere during left turn tasks. These results validate the central functional role of the left prefrontal cortex in steep turn tasks from both feature importance and inter-group difference perspectives.

**Figure 10:**
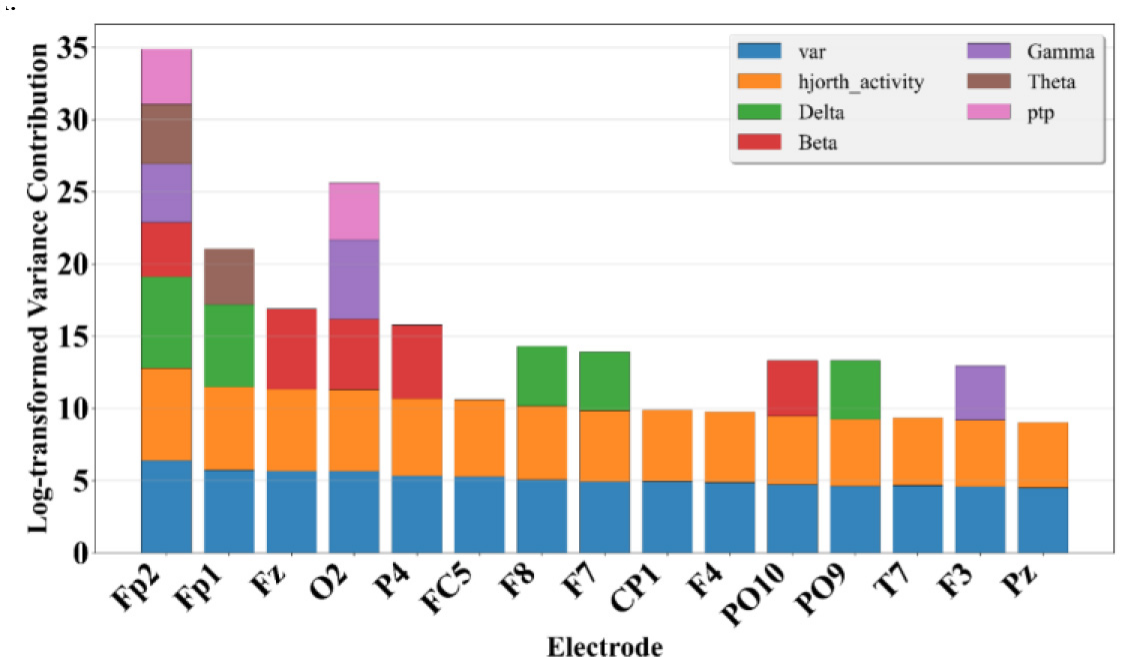
Important features sorted by electrode and variance.

To avoid conflating predictive utility with signal dispersion, we intentionally present two complementary views. Figure 9 (RF-based importance) quantifies model-dependent discriminative contribution to classification, whereas Figure 10 provides a model-agnostic variance decomposition of the selected feature pool. The two views are partially convergent (frontal/frontopolar channels remain prominent) but not identical, as expected, because high variance does not necessarily imply high discriminative power. This complementary analysis improves interpretability and prevents over-attribution from a single ranking criterion. Figure 10 shows the electrode-wise stacked distribution of variance contributions (log-transformed) for the selected feature subset. Quantitatively, among the 80 selected features (top 10% variance-ranked), 47 are located on the top 15 electrodes, and 33 are outside this set (coverage: 58.75%), indicating a mixed pattern of concentration and distribution rather than extreme localization.

At the channel level, Fp2 contributes the largest variance, followed by Fp1, Fz, O2, P4, and FC5. The log-scale view highlights that a subset of electrodes accounts for a larger share of transformed variance, while many other channels still contribute non-negligible information. At the feature-type level, the dominant components are var and hjorth_activity, which are consistently dominant across nearly all top electrodes in the transformed space. Conversely, rhythm-specific terms (Delta/Beta/Gamma/Theta) and ptp appear as secondary contributors and are concentrated in a subset of sites. This pattern suggests that under steep-turn conditions, broad-amplitude fluctuation descriptors and activity-level descriptors capture the main between-trial dispersion, whereas band-limited components provide more localized complementary information. Spatially, the high-variance electrodes are distributed across frontal/frontopolar (Fp2/Fp1/Fz), central-parietal (CP1/P4), and occipital-related sites (O2/PO9/PO10), suggesting that the variance structure may reflect a multi-component process involving executive attentional regulation, sensorimotor coordination, and visual-reference processing. Compared with the RF-based importance results, the variance map provides model-agnostic support for the stability of key spatial-feature patterns. That is, RF importance reflects direct predictive contribution to class separation, whereas variance ranking reflects intrinsic signal dispersion.

### 4.4 Top 10 important feature analysis for left/right steep turns

#### 4.4.1 Top 10 ranked by RF

The mean values of all valid subjects for the top 10 most significant features in the importance ranking were selected to explore differences when pilots perform left and right turns, as shown in Table 3. Overall, under left-turn conditions, the left brain regions exhibit higher high-frequency energy, complexity, and zero-crossing rates, while during right turns, the right brain regions demonstrate predominant energy in the Theta/Alpha frequency bands. Although the absolute differences in mean values for most features between left- and right-turn tasks were numerically small, these subtle variations arose from standardized EEG signal metrics, where standardization eliminated inter-subject variability in baseline EEG amplitude. This preprocessing ensured that the observed minor differences reflected genuine task-related neural activity modulation rather than physiological variability across individuals.

**Table 3:** Comparison of the top 10 important features between left and right turn.

Features	Significance	Importance	Left turn		Right turn		Difference (left turn-right turn)
			Mean	Std	Mean	Std	Mean
FC2_Alpha_abs_power	0.0065	0.0025	3.74	3.15	4.08	3.66	−0.34
F4_Gamma_rel_power	0.0012	0.0021	0.18	0.14	0.17	0.15	0.01
FC6_perm_entropy	0.0004	0.0021	0.79	0.01	0.78	0.01	0.01
FC6_svd_entropy	0	0.0021	0.67	0.09	0.66	0.09	0.01
P3_svd_entropy	0.0002	0.0019	0.63	0.07	0.62	0.07	0.01
F4_zcr	0	0.0019	71.64	24.56	68.14	26.84	3.50
Fp2_zcr	0.0313	0.0019	67.45	24.69	65.58	26.65	1.87
T7_Theta_abs_power	0.0005	0.0019	5.57	4.86	6.35	7.50	−0.78
F4_samp_entropy	0.0003	0.0019	1.22	0.18	1.18	0.22	0.04
Fz_hjorth_mobility	0.0397	0.0019	0.36	0.13	0.35	0.13	0.01

The absolute power in the Alpha frequency band (FC2_Alpha_abs_power) during left turns (3.74) is significantly lower than during right turns (4.08), reflecting Alpha event-related desynchronization (ERD) in the right motor cortex (near FC2). Alpha ERD refers to the phenomenon where Alpha power in the contralateral motor cortex decreases during motor preparation (Pfurtscheller and da Silva, 1999). Left-turn tasks involve activation of the left motor cortex, resulting in decreased Alpha power in the contralateral region (right FC2 area). Although the absolute mean difference for this feature was small (Δ = −0.34), the change showed a consistent directional trend across all subjects. Compared to the magnitude of absolute differences, this within-subject consistency in variation constitutes the core factor driving classification performance. The Random Forest algorithm preferentially selects features with stable discriminative trends across samples, even when their absolute differences are small, rather than features with large numerical differences but inconsistent variation patterns.

The relative power in the Gamma frequency band (F4_Gamma_rel_power) during left turns (0.18) is higher than during right turns (0.17), reflecting Gamma event-related synchronization (ERS) in the left motor-related brain region (near F4). Gamma ERS indicates that Gamma frequency synchronization is associated with local neuronal cooperation and fine motor control (Tallon-Baudry et al., 1997). The increased Gamma power in the F4 region during left turns reflects enhanced neural synchronization in the left hemisphere during left-turn tasks. For relative power metrics normalized by total whole-brain EEG power, a difference of 0.01 corresponds to a 5.6% proportional change relative to the left turn mean. This proportional change carries significant biological meaning, as Gamma-band oscillations are highly sensitive to motor planning processes, where even subtle proportional fluctuations can encode distinct motor intentions.

The absolute power in the Theta frequency band (T7_Theta_abs_power) during left turns (5.57) is lower than during right turns (6.35), suggesting enhanced Theta energy in the left temporal region (T7) during right turns. Changes in theta frequency power are typically associated with cognitive load and attention concentration (Gao et al., 2023), usually increasing when processing complex tasks. During right turns, pilots require higher attention and cognitive control to coordinate multi-control surface operations and integrate visual information, resulting in higher Theta power. Notably, the absolute power in the Theta band at electrode T7 exhibited relatively large standard deviations (left turn: 4.86; right turn: 7.50), indicating substantial inter-subject variability in baseline levels for this metric. However, the directional difference, in which left-turn power was consistently lower than right-turn power, remained consistent across all subjects. The RF model effectively captured this consistent directional pattern embedded within the variability, thereby assigning high importance to this feature despite its small absolute mean difference.

The perm_entropy of FC6, svd_entropy of FC6 and P3, and samp_entropy of F4 all show higher entropy values during left turns, indicating more complex EEG patterns in these brain regions during left-turn tasks. Increased entropy values (perm_entropy, svd_entropy, and samp_entropy) correspond to increased complexity of EEG patterns (Jorgsson, 2023), reflecting multi-regional information integration processes. In motor imagery tasks, brain regions involved in motor planning (such as the frontal and parietal lobes) exhibit increased entropy, consistent with the higher entropy in FC6 and P3 regions during left turns. Entropy-based metrics quantify the regularity of EEG time series, where a difference of 0.01 in entropy values signifies a substantial reduction in signal predictability. In brain-computer interface and motor intention classification tasks, entropy-based features possess unique value, as they encode the spatiotemporal complexity of neural networks rather than simple amplitude variations. Consequently, the subtle yet consistent differences in entropy values between left and right steep turn tasks can provide the model with robust discriminative information.

The ZCR of F4 and Fp2 is higher during left turns, reflecting richer high-frequency components in frontal EEG during left turns (ZCR is positively correlated with signal frequency). ZCR is positively correlated with signal frequency, and increased ZCR suggests an increased percentage of high-frequency components (AlSharabi et al., 2023). During motor preparation, high-frequency activity in frontal regions is enhanced, resulting in higher ZCR in F4 and Fp2 regions during left turns. Unlike normalized metrics, the zero-crossing rate showed larger absolute differences between left- and right-turn tasks (difference of 3.50 for F4 and 1.87 for Fp2), thereby directly enhancing its importance in the classification model. For features with smaller mean differences (such as permutation entropy at FC6), their synergistic interaction with other complementary features (such as sample entropy at F4 and singular value decomposition entropy at FC6) further strengthened classification performance. The model does not rely on a single highly discriminative feature; rather, it achieves accurate classification by leveraging the collective discriminative capacity of multiple weakly informative features.

The Hjorth mobility (signal variation rate indicator) of Fz is higher during left turns, reflecting more active EEG signal changes in the Fz region during left-turn tasks. Hjorth mobility measures the rate of signal change, and increased mobility reflects more active EEG activity (Talaat et al., 2025). During the motor preparation phase, the prefrontal cortex participates in motor decision-making, with enhanced signal dynamics, consistent with the results of higher mobility in the Fz region during left turns. Hjorth activity is a dimensionless metric that reflects the relative rate of signal variation; a difference of 0.01 indicates a 2.8% increase in signal dynamics in this region during the left turn task. This relative change is critical for distinguishing between left and right-turn motor intentions, as it reflects the differential activation state of the prefrontal cortex during the planning of asymmetric motor actions.

The EEG feature differences between left-turn and right-turn tasks reflect a comprehensive manifestation of brain functional lateralization (contralateral motor cortex regulation), neural synchronization (Gamma ERS), information integration (entropy increase), and dynamic activation (ZCR, Hjorth), consistent with classical theories of motor-related EEG (ERD/ERS, complexity changes, etc.). In summary, the high importance of these features despite small mean differences in the classification model can be attributed to three core factors: (1) Inter-subject consistency in the direction of differences, enabling the model to learn stable discriminative patterns; (2) Biological relevance of the features, whereby subtle numerical changes correspond to neurophysiological processes with clear physiological significance (such as ERD/ERS and neural synchronization); (3) Complementarity among multiple features, where the synergistic contribution of multiple weakly discriminative features substantially enhances the model's capacity to distinguish between left and right turn intentions. Furthermore, the dynamic regulatory mechanisms of Gamma synchronization (Spooner et al., 2021), the association between entropy values and cognitive complexity (Cofré and Destexhe, 2025), clinical validation of EEG features (Qi et al., 2025), and brain functional lateralization and dynamic activation (Bao et al., 2025) provide new perspectives for the neural encoding of motor intentions.

Furthermore, integrating neuropsychological theory, the intergroup differences in the top 10 features observed in this study are strongly indicative of differential neural encoding between left and right steep turn tasks. The elevated high-frequency energy and complexity in the left hemisphere during left turn tasks essentially represent the neural signatures of motor planning and fine control executed by the left motor cortex and prefrontal cortex. Gamma-band event-related synchronization (ERS), a marker of local neuronal cooperation, directly supports the precise execution of left turn maneuvers. Conversely, the predominance of theta/alpha-band energy in right-hemisphere regions during right-turn tasks is associated with higher cognitive load. Increased theta-band power typically accompanies elevated working memory load and enhanced attentional regulation, corroborating findings from NASA-TLX ratings indicating increased temporal demand and effort during right-turn tasks.

#### 4.4.2 Top 10 ranked by variance

To reduce model dependency, we further analyzed the top 10 variance-ranked features as a model-agnostic complement to RF-based importance. Variance ranking captures signal dispersion and potential separability, whereas RF importance reflects classifier-specific predictive contribution. Therefore, these two analyses characterize different but complementary aspects of feature utility.

As shown in Table 4, the variance-ranked set is dominated by amplitude-dispersion descriptors (e.g., var, hjorth_activity) and low-frequency power (Delta_abs_power), primarily from frontal/frontopolar electrodes (Fp2, Fp1, and Fz) and occipital O2. Across these features, right-turn means are generally higher than left-turn means (negative L–R values), indicating stronger large-scale amplitude fluctuations during right turns. This trend is consistent with our interpretation of higher task demand in right turns. Importantly, variance ranking and RF ranking are not expected to yield identical top features. Variance preferentially highlights high-dispersion features, while RF prioritizes features with stable discriminative patterns under cross-validation. The convergence of both analyses at the regional level (frontal/frontocentral dominance) strengthens the robustness of our main conclusion: left–right steep turns are associated with reliable directional differences in neurophysiological signatures, supported by complementary evidence from statistical testing, model-agnostic variance analysis, and model-based feature importance.

**Table 4:** Comparison of the top 10 variance-ranked features between left-turn and right-turn.

Feature	Variance importance	Left turn Mean	Left turn Std	Right turn Mean	Right turn Std	Difference (left-right)
Fp2_var	2,491,778	367.4135	1,647.666	422.2003	1,507.765	−54.7868
Fp2_hjorth_activity	2,482,054	366.6959	1,644.448	421.3757	1,504.82	−54.6798
Fp2_Delta_abs_power	2,110,233	157.0616	1,475.945	185.6237	1,430.168	−28.5622
Fp1_var	552,538.5	277.4946	722.7545	317.5773	763.0958	−40.0827
Fp1_hjorth_activity	550,382.3	276.9526	721.3428	316.957	761.6054	−40.0044
Fp1_Delta_abs_power	517,908.5	121.292	672.5599	147.0177	763.6073	−25.7256
Fz_var	463,973.5	184.4611	678.33	192.9473	684.382	−8.48621
Fz_hjorth_activity	462,162.9	184.1008	677.0051	192.5704	683.0453	−8.46964
O2_var	445,420.9	155.6214	663.1365	179.1977	671.8386	−23.5763
O2_hjorth_activity	443,682.7	155.3175	661.8413	178.8477	670.5264	−23.5302

### 4.5 Research limitations

This study has several limitations that should be considered when interpreting the findings. For clarity, we summarize the key limitations as follows:

- Simulator-based setting. All experiments were conducted in a desktop flight simulator. Although this setting supports controlled and repeatable data collection, it may not fully replicate real-world flight factors such as atmospheric turbulence, vestibular stimulation, and operational stressors. Therefore, the current results should be regarded as simulator-based evidence pending real-flight validation.
- Fixed task order (left first, right second). The experimental protocol used a fixed sequence in which the left steep turn always preceded the right steep turn. As a result, potential order effects (e.g., adaptation, learning, fatigue, or time-on-task) are inherently confounded with directional differences (Christensen et al., 2012). This design constraint limits causal attribution of left–right effects solely to workload-related mechanisms (Li et al., 2020).
- Lack of objective behavioral/perceptual covariates. The present study did not record objective covariates (e.g., control-input intensity, flight-performance deviations, or visual-scanning metrics). Consequently, we cannot statistically isolate whether the observed EEG differences reflect MWL-specific effects or a composite of cognitive, motor-execution, and perceptual demands associated with directional asymmetry.
- Homogeneous participant cohort (right-handed men cadets). Participants were exclusively right-handed men flight cadets, reflecting the institutional availability of the training cohort during the study period. Nevertheless, this sample composition restricts generalizability. Sex and handedness can influence EEG characteristics and feature distributions, and thus may affect both identified biomarkers and model performance. Caution is warranted when extending these findings to broader pilot populations (Darvishi-Bayazi et al., 2023).

To address the above limitations in a reproducible manner, future studies will: (i) adopt a counterbalanced design (randomized left/right order with standardized rest intervals) and include a control condition to disentangle direction from order/time-on-task; (ii) record objective covariates capturing control effort (integrated absolute control-input intensity; deflection magnitude/rate for rudder/aileron/elevator), flight performance (RMSE and time-out-of-tolerance for bank angle, altitude, airspeed, and heading), and visual demand (eye-tracking fixation dispersion, scanpath entropy, and instrument-vs.-outside dwell ratio); (iii) incorporate these covariates analytically via mixed-effects models or ANCOVA-style regression (subject as a random effect), and perform EEG feature residualization/partial-correlation analyses prior to classification, complemented by matched-condition evaluations (e.g., left vs. right turns within matched control-input intensity bins); and (iv) extend validation to multi-session reliability testing, real-flight replication, and broader recruitment including women pilots, varied handedness, and diverse experience levels, and explore dynamic EEG representations (e.g., time-resolved connectivity/state-space features) as well as multimodal fusion (EEG + eye tracking + HRV).

## 5 Conclusion

This study examined directional differences between left and right steep turns using subjective ratings and EEG-based machine learning, and provides simulator-based evidence that directional maneuvers are associated with distinguishable neurophysiological patterns.

First, NASA-TLX ratings indicated greater subjective demand in right turns than in left turns under the present simulator protocol. Second, EEG analyses revealed directional differences in spectral and complexity-related signatures, with left turns showing relatively stronger high-frequency activity/complexity patterns and right turns showing relatively stronger theta/alpha-related patterns. Third, among the six evaluated classifiers, tree-based models (LightGBM, XGBoost, and GB) achieved the most stable performance, and the best-performing configuration for LightGBM used a compact top 10% variance-ranked feature subset. *Post-hoc* interpretation combining RF-based importance and variance-based profiling suggested a convergent emphasis on frontal/frontocentral regions, while highlighting that predictive contribution and intrinsic dispersion represent complementary notions of feature utility.

Given the fixed task order, lack of objective covariates, simulator context, and homogeneous cohort, the present findings should be interpreted as a proof of concept demonstrating that directional steep turns are associated with separable EEG patterns and subjective demand differences, rather than definitive causal isolation of MWL. Future work will prioritize counterbalanced maneuver order, objective covariate logging (control-input, performance, and visual-scanning metrics), and covariate-aware modeling (mixed-effects/regression and residualization strategies) to disentangle directional effects from order, motor execution, and perceptual demand. Further steps will include multi-session reliability testing, real-flight replication, and broader participant recruitment to improve external validity and support translation toward lightweight workload monitoring in training-oriented aviation settings.

## Data Availability

The raw data supporting the conclusions of this article will be made available by the authors, without undue reservation.

## Footnotes

1
https://pilotinstitute.com/how-to-make-perfect-steep-turns/
